# Solid-State Membrane Sensors Based on Man-Tailored Biomimetic Receptors for Selective Recognition of Isoproturon and Diuron Herbicides

**DOI:** 10.3390/membranes10100279

**Published:** 2020-10-12

**Authors:** Ayman H. Kamel, Abd El-Galil E. Amr, Mohamed A. Al-Omar, Abdulrahman A. Almehizia

**Affiliations:** 1 Department of Chemistry, Faculty of Science, Ain Shams University, Cairo 11566, Egypt; 2 Pharmaceutical Chemistry Department, Drug Exploration & Development Chair (DEDC), College of Pharmacy, King Saud University, Riyadh 11451, Saudi Arabia; malomar1@ksu.edu.sa (M.A.A.-O.); mehizia@ksu.edu.sa (A.A.A.); 3 Applied Organic Chemistry Department, National Research Center, Dokki 12622, Giza, Egypt

**Keywords:** phenylurea herbicides, isoproturon, diuron, solid-contact, potentiometric sensors, molecularly imprinted polymers (MIPs)

## Abstract

Solid-contact ion-selective electrodes (SC-ISEs) have shown great potential for routine and portable ion detection. The introduction of nanomaterials as ion-to-electron transducers and the adoption of different performance-enhancement strategies have significantly promoted the development of SC-ISEs. Herein, new solid-contact ion-selective electrodes, along with the implementation of multiwalled carbon nanotubes (MWCNTs) as ion-to-electron transducers and potassium tetrakis (p-chlorophenyl) borate (KTpClB) as lipophilic ionic additives, were presented for the detection of isoproturon (IPU) and diuron (DU) herbicides. Molecularly imprinted polymers (MIPs), with special molecule recognition properties for isoproturon (IPU) and diuron (DU), were prepared, characterized, and introduced as sensory recognition materials in the presented electrodes. Sensors revealed a near-Nernstian response for both isoproturon (IPU) and diuron (DU) with slopes of 53.1 *±* 1.2 (*r*^2^ = 0.997) and 57.2 *±* 0.3 (*r*^2^ = 0.998) over the linear ranges of 2.2 × 10^−6^–1.0 × 10^−3^ M and 3.2 × 10^−6^–1.0 × 10^−3^ M with detection limits of 8.3 × 10^−7^ and 1.4 × 10^−6^ M, respectively. The response time of the presented sensors was found to be <5 s and the lifetime was at least eight weeks. The sensors exhibited good selectivity towards isoproturon (IPU) and diuron (DU) in comparison with some other herbicides, alkali, alkaline earth, and heavy metal ions. The presented sensors were successfully applied for the direct determination of isoproturon (IPU) and diuron (DU) in real water samples.

## 1. Introduction

Herbicides account for about 50% of agrochemical demand. The danger of these chemicals lies in their prolonged use and the risks of their presence in crops and soil. These materials are transferred to surface water and groundwater due to the washing and leaching operations of agricultural soil [1]. Phenylurea herbicides are selective herbicides that are commonly used in agriculture, either alone or in combination with other pesticides, to treat soil before emergence. Due to their polar nature, potential leakage from the surface into water supplies and water reserves, together with the emergence of the potentially toxic degradation and metabolic products, poses a risk to human health [2,3]. Duron is considered to be a persistent toxic herbicide in the phenylurea herbicide family. The European Commission has listed it as a priority hazardous substance. Its half-life is more than 300 days, so its survival in soil without decomposition is a dangerous reservoir, especially as it is used to control emergency situations in crop and noncrop areas [4]. Diuron has high persistence as well as genotoxicity, so there are many reported strategies aimed to provide its uptake and degradation in various ways [5]. Isoproturon also belongs to the family of phenylurea pesticides, which exhibit similar properties such as stability, persistence, and solubility in water. The uncontrolled use of these dangerous compounds poses a severe risk to the environment as well as aquatic organisms. This raises major concerns due to their slow biodegradation and their genetic and environmental toxicity [6]. 

Various analytical techniques have been indexed for the determination of different phenylurea compounds [7,8,9,10,11,12]. These reported methods required complex procedures and nonportable equipment. In addition, they need prior derivatization or extraction steps and involve expensive equipment and chemicals. All of these drawbacks do not enable the use of such methods in the routine analysis of such herbicides. 

Among these techniques, electrochemical methods can be very attractive because of their fast response, low cost, high selectivity, suitability for on-site analysis, and reproducibility [13,14]. Moreover, these techniques can allow multicomponent quantification, resulting in rapid analysis that is time-saving and has an economic return and advantages. The few reported works available in the literature have been monitored on the basis of the electrochemical determination of phenylurea herbicides [15,16,17,18]. Among all electrochemical techniques, potential-based transduction “potentiometry” is potentially the most preferred technique. It presents good advantages such as quick and analysis, high sensitivity, cost-effectiveness, simplicity in use, and response reliability [19,20,21].

Solid-contact potentiometric sensors, especially, are considered attractive devices for a very wide range of practical applications [22,23,24,25]. In comparison with liquid-contact electrodes, solid-contact sensors are cost-effective, simpler to use and transport, and more mechanically resistant. Moreover, they are pressure resistant, can work in any position, are easy to back and seal, and allow miniaturization for portable devices. Unfortunately, direct contact between the electronic conductors and the ion-sensing membranes (ISM) leads to a large potential deviation due to the high resistance of the charge transfer [26]. To overcome these problems, an intermediate layer of conductive polymers or nanomaterials with mixed ion and electronic conductivity should be inserted between the ISM and the electronic conductor [27]. This intermediate layer should be hydrophobic to prevent the formation of a water layer on the interface and be resistant to the effects of CO_2_, pH, or light sensitivity [28,29].

For years, molecular imprinting technology has been a successful tool for preparing polymeric materials characterized by their high selectivity and affinity. The synthesis of molecularly imprinted polymers (MIPs) involves the assembly of monomers around a template molecule, followed by polymerization in the presence of a cross-linker and initiator. After template removal, specific sites for this template molecule are found with respect to both shape and chemical function, allowing for subsequent recognition of the template. Such synthetic receptors are characterized by their physical durability, high strength, resistance to high temperatures and pressure, and high stability with acids, bases, metal ions, and organic solvents. MIPs have been used in a wide variety of applications. These applications include catalysis processes [30], identification and determination of toxins in food matrices [31,32], solid-phase extraction (SPE) [33], drug delivery [34,35], biological antibody and receptor systems [36,37], and chemical sensor devices [38,39,40]. 

The integration of MIPs into potentiometric electrodes provide good features such as high selectivity and sensitivity, low detection limits, as well as easy miniaturization and automation. These synthetic receptors have been extensively used to quantify various organic and inorganic ions [41,42,43,44,45,46]. Until now, no potentiometric sensors for the determination of either isoproturon or diuron herbicides have been reported.

In the present work, we describe, for the first time, sensitive potentiometric sensors for the determination of isoproturon and diuron herbicides. The sensors are based on the dispersion of artificially imprinted receptors for these templates in 2-nitrophenyloctyl ether (o-NPOE) and embedded in a polyvinyl chloride (PVC) matrix. MWCNTs were used as solid-contact transducers and potassium tetrakis (p-chlorophenyl) borate (KTpClB) as a lipophilic ionic additive. The electrochemical performance characteristics of the proposed electrodes were evaluated for the simple, fast, selective, and cost-effective analysis of diuron and isoproturon in different wastewater samples collected from agricultural resources.

## 2. Experimental

### 2.1. Equipment

All potentiometric measurements were performed at room temperature (25 ± 1 °C) using a digital pH/mV meter (PXSJ-216, INESA Scientific Instrument Co., Ltd, Shanghai, China). An Ag/AgCl/KCl (3 M) double-junction reference electrode (Orion 90-02) filled with 0.1 M CH_3_COOLi in its outer compartment was used in conjunction with the proposed ion-selective electrode (ISE) to construct the electrochemical cell. A combined glass/pH electrode (Orion 81-02) was used for all pH measurements. The Milli-Q PLUS reagent-grade water system (Millipore, Burlington, MA, USA) (18.2 MΩ·cm specific resistance) was used for obtaining deionized water. Glassy carbon (GC) rods 3 mm in diameter were supplied from HTW GmbH. All spectrophotometric measurements were carried out using the Shimadzu UV-1601 (PC, Osaka, Japan) spectrophotometer. The controlled three-electrode system was used for all chronopotentiometric and impedance measurements. The cell assembly included a Pt wire as an auxiliary, and a single-junction Ag/AgCl (3 M) KCl as a reference electrode. 

### 2.2. Reagents and Materials

**Figure 1:**
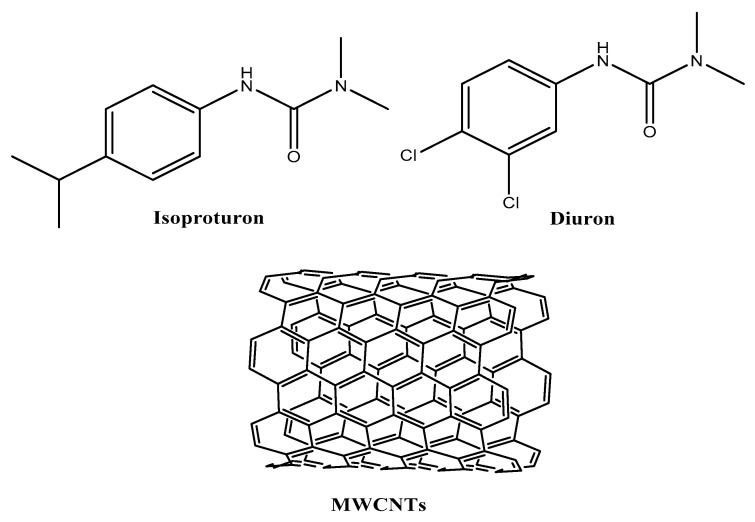
Chemical structure of the studied compounds.

All reagents were of analytical grade and used as received without further purification. High-molecular-weight polyvinyl chloride (PVC), tetrahydrofuran (THF), potassium tetrakis (p-chlorophenyl) borate (KTpClB), multiwalled carbon nanotubes (MWCNTs, >95% purity) with 6–9 nm in diameter and 5 µm in length, tetradodecylammonium tetrakis (4-chlorophenyl) borate (ETH500), and o-nitrophenyl octyl ether (o-NPOE) were obtained from Sigma-Aldrich (St. Louis, MO, USA). Methacrylic acid (MAA), ethylene glycol dimethacrylate (EGDMA), and benzoyl peroxide (BPO) were obtained from Fluka (Ronkonkoma, NY). Isoproturon (IPU) and diuron (DU) (see Figure 1 for chemical structures) were obtained from Riedel-de Haën (Seelze, Germany). 

The herbicide stock solutions (10^−3^ M) were prepared by dissolving an appropriate amount of the compound in methanolic solution and kept in the dark at −5 °C. Working solutions were prepared daily by diluting the stock solution with Britton–Robinson (BR) buffer (0.04 M boric acid, 0.04 M phosphoric acid, and 0.04 M acetic acid). The pH was adjusted with 0.2 M NaOH, covering the pH range from 2.0 to 7.0.

### 2.3. Synthesis of Host-Tailored Polymers

For the preparation of MIPs, 0.5 mmol of the template (either isoproturon or diuron) was mixed with 3.0 mmol of the functional monomer MAA in a glass tube and left for 1 h. Then, 3.0 mmol of the cross-linker EGDMA was added to the mixture followed by 0.3 mmol of the initiator BPO. All components were mixed together with 15 mL acetonitrile. Following this, the mixture was sonicated for 10 min until complete dissolution. The reaction mixture was degassed with N_2_ gas for 15 min, and placed in an oil bath for 18 h at 70 °C. The obtained polymeric beads were ground and washed with methanol to remove all unreacted species. Extraction of the template molecule was carried out via soxhlet using methanol/acetic acid (8:2, *v*/*v*). For complete removal of the template molecule from the MIP skeleton, the washing solution was measured spectrophotometrically at *λ_max_* = 245 and 250 nm to check the existence of either isoproturon or diuron, respectively. The particles were repeatedly washed until the herbicide was no longer detected. The resulting polymeric beads were left to dry at room temperature prior to use. Nonimprinted polymers (NIPs) were synthesized in a similar way as mentioned above with the exclusion of the template from the procedure.

### 2.4. Binding Experiments

Binding experiments were performed by inserting 20.0 mg of either MIP or NIP beads in contact with 10.0 mL of either IPU or DU solutions in the concentration range of 0.02–0.5 mM. The heterogeneous mixture was shaken for 12 h at an ambient temperature followed by centrifugation (3000× *g* rpm, 10 min) for solid-phase separation. The remaining concentration of the herbicide in the supernatant solution was determined using UV spectrophotometry measurements at the wavelength corresponding to the herbicide detected. The amount of each herbicide bound to the polymeric beads was evaluated after subtracting the free herbicide concentration from its initial value.

### 2.5. Sensor Preparation 

Glassy carbon (GC) disks with a diameter of 3 mm and a height of 5 mm were used as electronic conductors for solid-contact ISEs. The GC disks were carefully polished with 0.3 µm Al_2_O_3_ slurries, sonicated in ethanol for 15 min, and rinsed with water. The disks were washed with ethanol and dried in air. A piece of matched PVC tube (1 cm long, 5 mm i.d., and 8 mm o.d.) was tightly inserted at the distal end of the electrodes. The ISM cocktail (total mass of 112 mg in 2.0 mL THF) was prepared after dispersing MIP or NIP particles (12 mg), KTpClB (1.0 mg), ETH 500 (1 mg), o-NPOE (49.0 mg), and PVC (49.0 mg) together with 1 mg/mL of the MWCNTs in THF. The mixture was sonicated for 30 min until a uniform solution was obtained. A 100 µL volume of the membrane cocktail covered the GC disks via drop-casting, and the solvent was left to be evaporated overnight at room temperature. A uniform composite layer was obtained and strongly adhered to the GC surface. The coated-wire electrodes (CWEs) were also prepared by the same steps as mentioned above but without the addition of a MWCNT layer. All solid-contact-selective electrodes were, firstly, conditioned in 10^−4^ M of either IPU or DU solutions for 1 day; then, they were conditioned in 10^−8^ M of IPU or DU solutions for 2 days. A 50 mM BR buffer (pH = 3) was used to adjust the pH of all working solutions. 

### 2.6. Analytical Applications

Agriculture wastewater samples were collected from different areas in Giza, Egypt. The samples were degassed in an ultrasonic bath. A 10 mL volume of the sample solution was spiked with different aliquots of IPU and DU within a concentration range of 0.5–10.0 µg/mL. The working sensors, in conjunction with the reference electrode, were immersed in the test solution. The potential reading was recorded and the concentration of the test solution was determined from the constructed calibration plot. 

## 3. Results and Discussion

**Figure 2:**
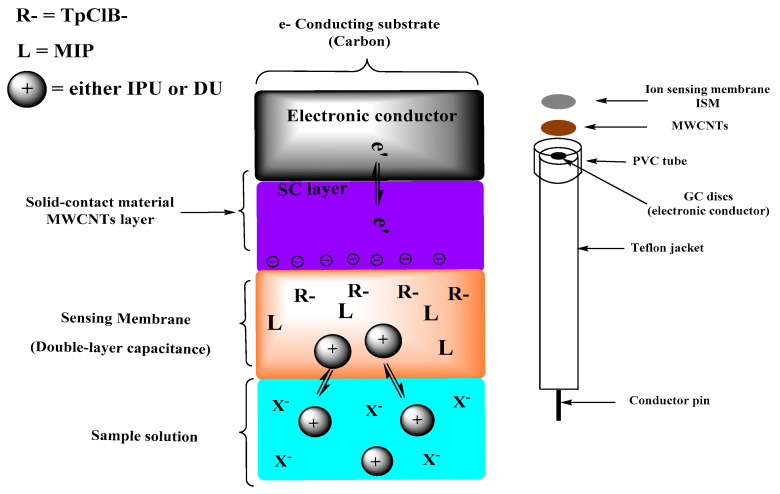
Response mechanism of the proposed solid-contact ion-selective electrodes (ISEs).

Herein, we aimed to present a fast, simple, reliable, and sensitive method of analysis based on MIPs for detecting isoproturon and diuron as phenylurea herbicides. For this purpose, we presented a solid-contact potentiometric sensor utilizing MWCNTs as ion-to-electron transducers. A schematic illustration for the proposed mechanism of transduction is shown in Figure 2.

### 3.1. Equilibrium Adsorption 

**Figure 3:**
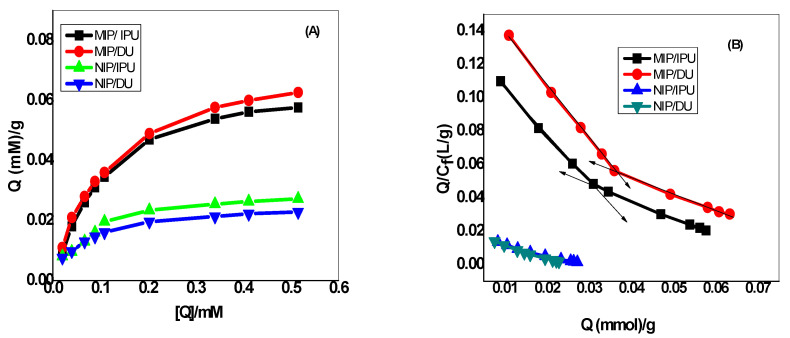
Binding isotherm (**A**) and Scatchard plot (**B**) for both isoproturon (IPU)- and diuron (DU)-imprinted polymers. *Q* = herbicide bound to 20.0 mg of the corresponding polymer; temperature = 25 °C; volume = 10.0 mL; binding time = 12 h.

Adsorption isotherms are an important tool to explain how small molecules can interact with the surfaces of adsorbents. In these isotherms, the concentration at the equilibrium of the bound adsorbate is plotted versus the free adsorbate concentration. In liquid-phase applications of MIPs, the adsorbate molecules interact with the binding sites in the solid-adsorbent MIP. After equilibrium is attained, the constant-free adsorbate concentration can be determined to construct the corresponding adsorption isotherm [47]. The static equilibrium adsorption experiments for the synthesized MIPs were performed using 0.02–0.5 mM as an initial concentration of either IPU or DU herbicides. The obtained adsorption isotherms are shown in Figure 3a. It was noticed that the ability of MIPs to adsorb their corresponding herbicide increases as the initial concentration of the herbicide increases. The adsorbitivity of the NIPs reached a saturation state for all tested herbicides when the initial concentration of the herbicide was beyond 0.08 mM. It was found that the binding affinity of MIPs towards either IPU or DU herbicides was bigger than that of the NIP beads. This can confirm the existence of selective active sites with a high affinity and specific recognition on MIPs. 

The Scatchard model was used to evaluate the binding characteristics of either MIPs or NIPs. The Scatchard equation can be described as [47]:

[*Q*]/[*C_f_*] = ([*Q_max_*] − [*Q*])/*K_d_* (1)

where *K_d_* is the dissociation constant, *Q_max_* is the maximum binding capacity, and [*C_f_*] is the equilibrium-free concentration of the substrate in the supernatant. As mentioned in Figure 3b, the binding sites of the prepared MIPs were heterogeneous. This can conclude the existence of two binding site classes with different affinities in the range of the different concentrations. This can be attributed to the presence of various modes of interaction between the template molecule and the functional monomer. These modes of interaction can form different kinds of complexes with different components that lead to the formation of different binding sites with different properties after polymerization. The two sections of the linear plot in Figure 3b can fit the data. At the higher-affinity binding sites, the dissociation constant *K_d_*_1_ and the apparent maximum amount *Q_max_*_1_ were found to be 0.344 and 0.31 mM and 0.046 and 0.053 mmol/g for MIP/IPU and MIP/DU, respectively. By the same treatment, *K_d_*_2_ and *Q_max_*_2_ for the lower-affinity binding sites were calculated to be 0.969 and 1.036 mM and 0.077 and 0.093 mmol/g for MIP/IPU and MIP/DU, respectively. In contrast, the *K_d_* and *Q_max_* values were found to be 1.583 and 1.288 mM and 0.028 and 0.0245 mmol/g for NIP/IPU and NIP/DU, respectively. This indicates a high homogeneity of the binding sites present in NIP beads and shows a lower affinity towards the studied phenylurea herbicides than their corresponding MIPs.

### 3.2. Surface Morphology

**Figure 4:**
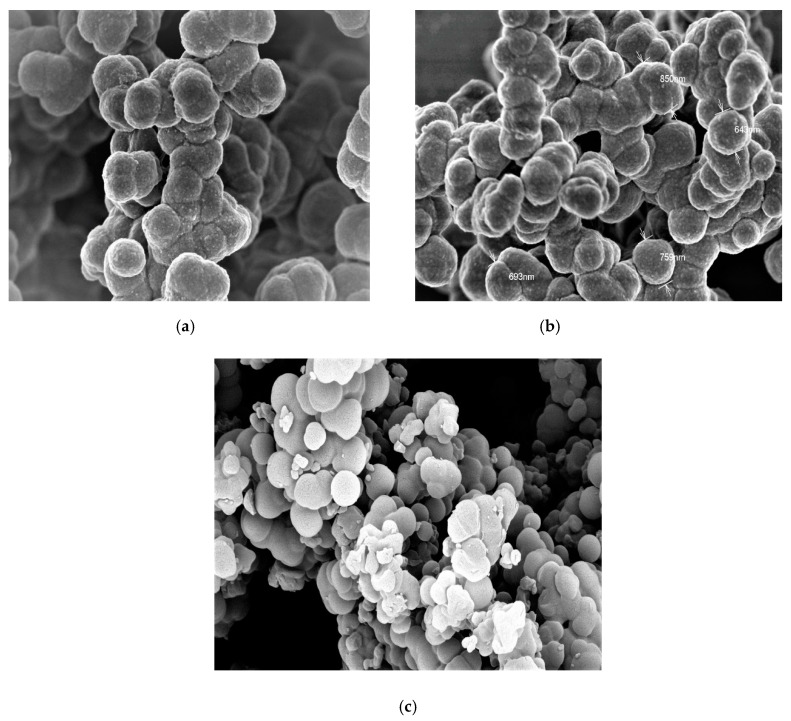
SEM images of (**a**) molecularly imprinted polymers (MIP)/IPU, (**b**) MIP/DU, and (**c**) nonimprinted polymer (NIP) beads. Conditions: scale bar = 2.00 µm, 3.0 kv; 27 mm x20.0kv.

The surface morphologies of both NIPs and MIPs after template removal were also examined and shown in the SEM micrographs (Figure 4). For MIPs, the beads were semispherical with an irregular shape and had a size ranging between 0.8 and 1.1 and 0.65 and 0.95 µm for both MIP/IPU (Figure 4a) and MIP/DU (Figure 4b), respectively. For NIPs, it was found that the beads were spherical, smooth, and uniformly shaped, with a size ranging between 1 and 2 µm, as mentioned in Figure 4c. This can be attributed to the absence of specific binding sites in the polymers. These morphological differences can confirm the presence of the imprinting process and the ability of MIPs to adsorb and uptake these phenylurea herbicides more than NIP beads.

### 3.3. ISE Performance Characteristics

The prepared MIPs were dispersed and introduced as sensing materials into a plasticized PVC membrane in the presence of the anionic additive KTpClB to prepare the presented electrodes. 

**Figure 5:**
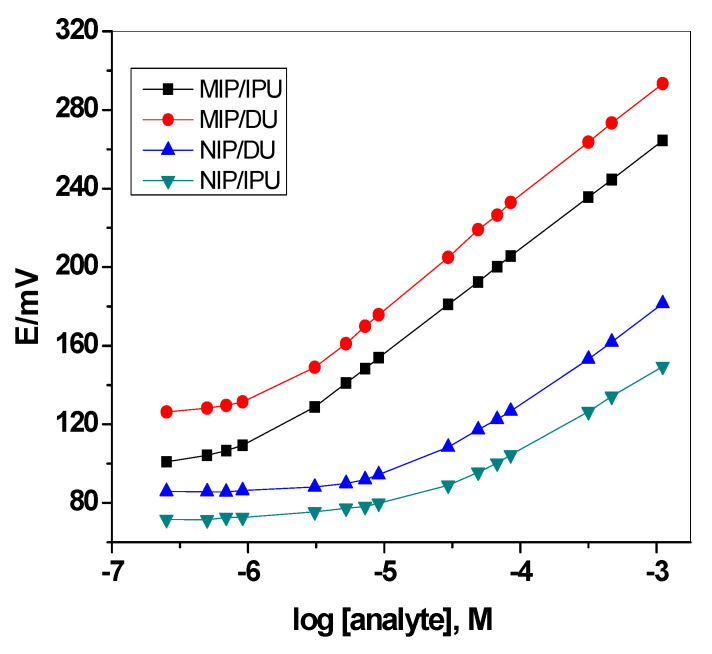
Calibration plots for all solid-contact ISEs in the Britton–Robinson (BR) buffer solution (pH = 3.0).

The potential-log [concentration] plots for MIP/IPU (Sensor 1) and MIP/DU (Sensor 2) are shown in Figure 5. The sensors exhibited a linear potentiometric response over the ranges of 2.2 × 10^−6^–1.0 × 10^−3^ M and 3.2 × 10^−6^–1.0 × 10^−3^ M with detection limits of 8.3 × 10^−7^ and 1.4 × 10^−6^ M and near-Nernstian slopes of 53.1 *±* 1.2 (*r*^2^ *=* 0.997) and 57.2 *±* 0.3 (*r*^2^ = 0.998) mV/decade for Sensors 1 and 2, respectively. Sensors based on NIPs (i.e., NIP/IPU (Sensor 3) and NIP/DU (Sensor 4) exhibited a linear potentiometric response within the ranges of 3.2 × 10^−5^–1.0 × 10^−3^ and 3.4 × 10^−5^–1.0 × 10^−3^ M with detection limits of 1.3 × 10^−5^ and 1.2 × 10^−5^ M and sub-Nernstian slopes of 39.8 ± 0*.7* (*r*^2^ = 0.999) and 47.2 *±* 0.9 (*r*^2^ = 0.998), for Sensors 3 and 4, respectively. The performance characteristics of the presented sensors were shown in Table 1.

To test method repeatability, the spread of results obtained for the concentration of either IPU or DU herbicides (i.e., 10 µg/mL) when measured within-day and between-days under different method conditions and sensor assemblies were calculated. The within-day data repeatability was found to be 0.6% and 0.8%, while between-day variability was found to be 1.1% and 0.9% for Sensors 1 and 2, respectively.

The standard deviations (σ_v_ ) of the measured potential (*n* = 5) were found to be 1.1 and 0.7 mV for the test solution of 0.1 mM for Sensors 1 and 2, respectively. It was found that the performance response characteristics of the electrodes did not record a remarkable change after the use of the presented electrodes for eight weeks. The validity of the proposed potentiometric method for determining each herbicide was evaluated after determining the range of linearity, detection limit, accuracy (recovery), within-day repeatability (precision, Cv_w_), between-day variability (Cv_b_), and sensitivity in terms of slope [48]. All data obtained were of six batches of each herbicide solution and were shown in Table 1.

**Figure 6:**
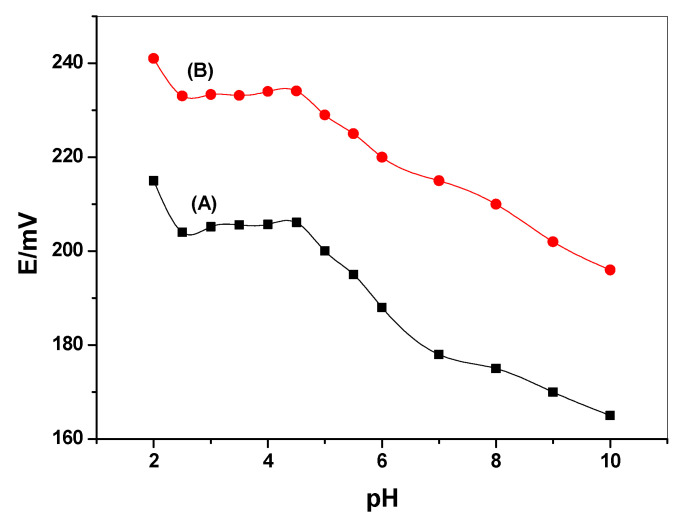
pH plot for (A) MIP(IPU)/multiwalled carbon nanotubes (MWCNTs)-ISEs and (B) MIP(DU)/MWCNTs-ISEs.

The influence of the pH on the potential response of the proposed sensors was tested using 10^−4^ M of the corresponding herbicide over the pH range of 2–10 (Figure 6). The adjustment of the pH of each solution was done using NaOH. The pH potential plots showed that the electrodes displayed constant potential readings over the pH range 2.5–4.5 for all presented sensors. A 50 mM BR buffer at pH 3 was used for all herbicide measurements. 

**Figure 7:**
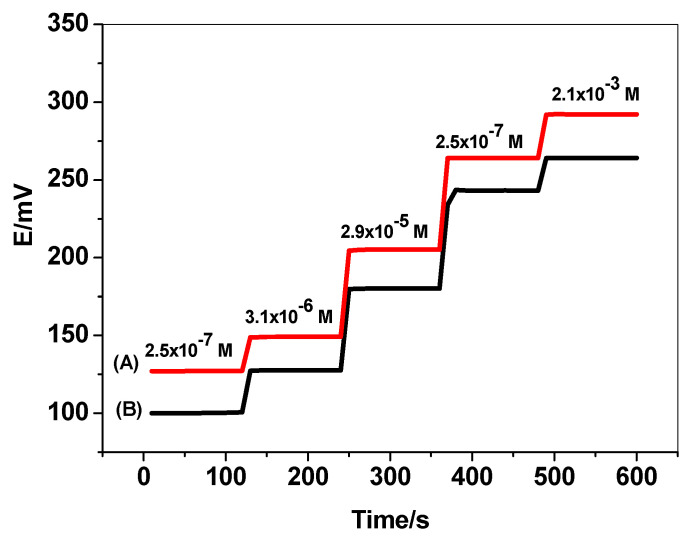
Time response for (A) MIP(IPU)/MWCNTs-ISEs and (B) MIP(DU)/MWCNTs-ISEs.

The time taken by the sensors to reach a steady-state potential within ±0.8 mV of the final equilibrium value was recorded after the successive immersion of the electrodes in a series of their corresponding herbicide solutions. Each had 10-fold differences, from low to high concentrations. The response time was <10 s for all herbicide solutions of concentrations in the linear range of calibration curves indicating the fast response of the electrodes (Figure 7). 

### 3.4. Sensors’ Selectivity

The selectivity behavior of the presented ISEs was evaluated using a modified separate solution method (MSSM) [49]. In brief, the electrodes were subjected to the less-discriminated ion. The potential at *a*_ion_ = 1 M was recorded from the extrapolation of the calibration plot of each interfering ion. The selectivity coefficient values were calculated for all sensors and are summarized in Table 2. The typical selectivity order of MIP/IPU- and MIP/DU-based sensors with a membrane plasticized with *o*-NPOE was: IPU > MU > FU > LU > DU > phenyl urea > phenylalanine > urea > NH_4_^+^ > K^+^ > Na^+^ > Ca^2+^ and DU ~ LU > FU > MU > IPU > phenyl urea > phenylalanine > urea > NH_4_^+^ > Na^+^ > K^+^ > Ca^2+^, respectively. The selectivity pattern in ISEs strongly depended on the recognition affinity of the ligand to the analyte under study. Therefore, the obtained selectivity order of the synthesized MIPs suggests that the mechanism of selectivity is mainly governed by stereo-specific and electrostatic aspects, where the lipophilic environment is dictated by the plasticizer.

### 3.5. Impedance and Chronopotentiometric Measurements 

**Figure 8:**
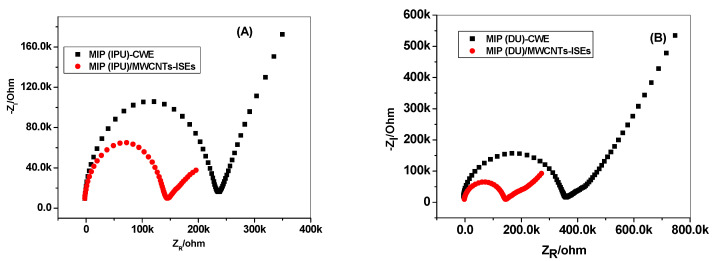
(**A**) Impedance plots of MIP (IPU)/MWCNTs-ISEs (red) and MIP (IPU)-coated-wire electrodes (CWEs) (black) in the 10^−4^ M IPU solution. (**B**) Impedance plots of MIP (DU)/MWCNTs-ISEs (red) and MIP (DU)-CWE (black) in the 10^−4^ M DU solution. Conditions: the frequency ranges from 100 kHz to 0.01 Hz and the disturbance amplitude is 10 mV.

Impedance and chronopotentiometric measurements were carried out to evaluate the performance of MWCNT transducers in the presented solid-contact ISEs. The impedance spectra of MIP (IPU)/MWCNTs-ISEs, MIP (IPU)-CWE, MIP (DU)/MWCNTs-ISEs, and MIP (DU)-CWE are shown in Figure 8. Large semicircles in the high-frequency region were obtained. These are related to the membrane’s bulk resistance. It was noticed that the diameter of the obtained semicircles decreased from 0.27 to 0.14 MΩ and from 0.36 to 0.15 MΩ after the insertion of MWCNTs, i.e., from MIP (IPU)/MWCNTs-ISEs to MIP (IPU)-CWE and from MIP (DU)/MWCNTs-ISEs to MIP (DU)-CWE, respectively. The contact resistance decreased after the addition of the MWCNT layer. In addition, the charge transfer at the solid-contact interface also improved [50]. At the low-frequency region, a large semicircle was noticed with both the MIP (IPU)-CWE and MIP (IPU)-CWE but none with the MIP (IPU)/MWCNTs-ISEs and MIP (DU)/MWCNTs-ISEs. This is due to the presence of a small double-layer capacitance and large transfer resistance at the interface between the ISM and the GC substrate in the electrodes with no MWCNT films. MWCNT film acts as an ion-to-electron transducer that increases the interface contact capacitance and greatly promotes the ion-electron transfer process.

To evaluate the double-layer capacitance of the MWCNTs transducer, which is related to the potential stability of the electrode, chronopotentiometric measurements were carried according to the protocol suggested by Bobacka [50]. The measurements were carried out after applying a ±1 nA current through a controlled three-electrode cell composed of the working electrode, a single-junction Ag/AgCl/KCl (1 M) reference electrode, and Pt wire as an auxiliary electrode. All were immersed in 10^−4^ M of the herbicide solution. 

**Figure 9:**
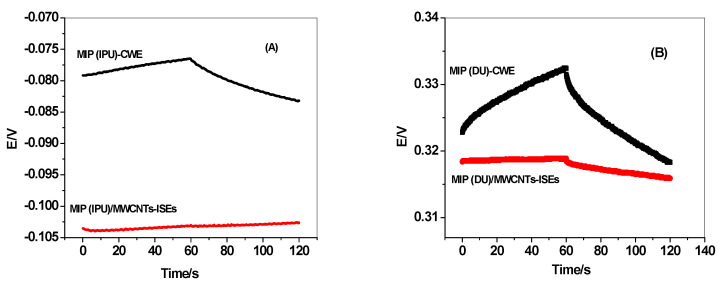
Chronopotentiometric plots of (**A**) MIP (IPU)/MWCNTs-ISEs (red) and MIP (IPU)-CWE (black) in the 10^−4^ M IPU solution. (**B**) MIP (DU)/MWCNTs-ISEs (red) and MIP (DU)-CWE (black) in the 10^−4^ M DU solution.

Chronopotentiometric plots for all the presented sensors are shown in Figure 9. The short-term potential stability for each electrode was evaluated from the slope (ΔE/Δt) of the *E-T* curve at longer times. The potential drift for MIP (IPU)/MWCNTs-ISEs, MIP (IPU)-CWE, MIP (DU)/MWCNTs-ISEs, and MIP (DU)-CWE was found to be 9.42, 103.4, 22.3, and 189.5 µV/s, respectively. The interfacial capacitance was also evaluated using the formula ΔE/Δt = i/C_L_. The obtained double-layer capacitances for MIP (IPU)/MWCNTs-ISEs, MIP (IPU)-CWE, MIP (DU)/MWCNTs-ISEs, and MIP (DU)-CWE were found to be 106.1, 9.67, 44.8, and 5.29 µF. The high electric capacity obtained after inserting the MWCNT layer in MIP (IPU)/MWCNTs-ISEs or MIP (DU)/MWCNTs-ISEs plays an essential role in resisting the external current’s polarization [51].

### 3.6. Analytical Application: Monitoring of Phenylureas in Water Samples

To test the applicability of the presented ISEs, they were used to determine IPU and DU herbicides in different wastewater samples collected from different agricultural sources. The water was mixed and spiked with 0.5–10.0 µg/mL phenylureas. The results of the potentiometric analysis conducted in steady-state showed recoveries ranging from 96.8 to 106.1% and 96.0 to 104% for MIP (IPU)/MWCNTs-ISEs and MIP (DU)/MWCNTs-ISEs, respectively. As shown in Table 3, the results obtained for the analysis of water samples presented good accuracy and demonstrated the applicability of the sensors for routine analysis without prior separation. 

## 4. Conclusions

A molecular imprinting technique was assigned to design isoproturon (IPU) and diuron (DU) host-tailored sensors for potentiometric transduction. Multiwalled carbon nanotubes (MWCNTs) were employed as ion-to-electron transducers and potassium tetrakis (p-chlorophenyl) borate (KTpClB) as a lipophilic ionic additive. The sensors revealed a near-Nernstian response for both isoproturon (IPU) and diuron (DU) with slopes of 53.1 ± 1.2 (*r*^2^ = 0.997) and 57.2 ± 0.3 (*r*^2^ = 0.998) over the linear ranges of 2.2 × 10^−6^–1.0 × 10^−3^ M and 3.2 × 10^−6^–1.0 × 10^−3^ M with detection limits of 8.3 × 10^−7^ and 1.4 × 10^−6^ M, respectively. The response time of the presented sensors was found to be <5 s and the lifetime was at least eight weeks. Simplicity in design, a short measurement time, good precision, high accuracy, high analytical throughput, and low detection limits were the advantages of these sensors. The sensors exhibited good selectivity towards isoproturon (IPU) and diuron (DU) in comparison with some other herbicides, alkali, alkaline earth, and heavy metal ions. The presented sensors were successfully applied for the direct determination of isoproturon (IPU) and diuron (DU) in real water samples. The proposed method is simple, of low cost, precise, accurate, and inexpensive regarding reagent consumption and the equipment involved. 

## Figures and Tables

**Table 1:** Potentiometric response characteristics of phenylureas sensors.

Parameter	MIP(IPU)/MWCNTs-ISEs (Sensor 1)	MIP(DU)/MWCNTs-ISEs (Sensor 2)	NIP/IPU (Sensor 3)	NIP/DU (Sensor 4)
Slope, mV/decade	53.1 ± 1.2	57.2 ± 1.2	39.8 ± 0.9	48.1 ± 0.6
Correlation coefficient, r^2^	0.997	0.998	0.998	0.998
Linear range, M	2.2 × 10^−6^–1.0 × 10^−3^	3.2 × 10^−6^–1.0 × 10^−3^	3.2 × 10^−5^–1.0 × 10^−3^	3.4 × 10^−5^–1.0 × 10^−3^
Detection limit, M	8.3 × 10^−7^	1.4 × 10^−6^	1.3 × 10^−5^	1.2 × 10^−5^
Working range, pH	2.5–4.5	2.5–4.5	2.5–4.5	2.5–4.5
Response time, s	<10	<10	<10	<10
Standard deviation (σ_v_), mV	1.1	0.7	1.2	0.9
Accuracy, %	99.1	98.8	97.6	96.8
Precision (CV_w_), %	0.6	0.8	0.6	0.5
Between-day variability (CV_b_), %	1.1	0.9	0.8	1.2

**Table 2:** Potentiometric selectivity coefficients (*Log K^pot^_i,B_*) of phenylureas PVC membrane sensors.

Interfering Ion, B	MIP (DU)/MWCNTs-ISEs	MIP(IPU)/MWCNTs-ISEs	NIP/DU	NIP/IPU
Isoproturon (IPU)	0	−3.1 ± 0.6	0	−3.3 ± 0.2
Diuron (DU)	−3.3 ± 0.6	0	−3.0 ± 0.1	0
Linuron (LU)	−2.9 ± 0.4	−0.7 ± 0.03	−1.7 ± 0.3	−1.4 ± 0.1
Fenuron (FU)	−2.8 ± 0.2	−2.2 ± 0.6	−1.8 ± 0.2	−1.7 ± 0.2
Methiuron (MU)	−2.7 ± 0.7	−2.9 ± 0.5	−2.0 ± 0.4	−2.8 ± 0.4
Phenylurea	−3.4 ± 0.6	−3.5 ± 0.3	−2.4 ± 0.2	−2.6 ± 0.4
Phenylalanine	−4.7 ± 0.8	−4.6 ± 0.9	−3.1 ± 0.3	−3.8 ± 0.2
Urea	−5.1 ± 0.2	−5.2 ± 0.1	−4.3 ± 0.5	−4.8 ± 0.4
NH_4_^+^	−5.6 ± 0.3	−5.5 ± 0.4	−4.6 ± 0.4	−5.2 ± 0.2
K^+^	−5.9 ± 0.2	−5.8 ± 0.3	−4.8 ± 0.1	−4.6 ± 0.5
Na^+^	−6.1 ± 0.1	−5.7 ± 0.9	−5.3 ± 0.3	−5.6 ± 0.2
Ca^2+^	−6.4 ± 0.2	−6.6 ± 0.1	−5.4 ± 0.6	−5.5 ± 0.1

Average of 3 measurements.

**Table 3:** Application of the proposed method to determination of phenylureas in spiked wastewater samples.

Sample	* Concentration of Phenylurea Herbicide, µg/mL				
	Amount Added	(IPU)/MWCNTs-ISEs		MIP (DU)/MWCNTs-ISEs	
		Found, IPU	Recovery, %	Found, DU	Recovery, %
Sample 1	0.5	0.53 ± 0.2	106.1	0.52 ± 0.3	104.0
Sample2	2.5	2.42 ± 0.4	96.8	2.53 ± 0.2	101.2
Sample3	5.0	4.87 ± 0.3	97.4	4.93 ± 0.4	98.6
Sample 4	10.0	9.7 ± 0.4	97.0	9.6 ± 0.5	96.0

* Average of 5 measurements.
